# Dynamic photobiology: A collection of experimental protocols for time‐resolved structural studies on light‐activated proteins

**DOI:** 10.1002/2211-5463.70303

**Published:** 2026-10-01

**Authors:** Sofia M Kapetanaki, Antoine Royant, Briony A Yorke

**Affiliations:** ^1^ MAX IV Laboratory Lund University Sweden; ^2^ Université Grenoble Alpes, CNRS, CEA, Institut de Biologie Structurale, Grenoble France; ^3^ European Synchrotron Radiation Facility France; ^4^ School of Chemistry University of Leeds UK

## Abstract

The study of proteins that sense and respond to light reveals how nature has harnessed light for function while also mitigating its damaging effects through finely tuned molecular mechanisms. Time‐resolved optical spectroscopy and time‐resolved X‐ray solution scattering and diffraction techniques are well‐positioned to probe functional transitions triggered by light in photoactive proteins. However, integrating both fields presents technical and conceptual difficulties for many researchers. This *FEBS Open Bio* ‘In the Limelight’ special issue, Dynamic Photobiology, showcases five research protocols that provide comprehensive and practical guidance for designing and analyzing time‐resolved experiments on proteins. The protocols cover studies performed in both solution and crystalline environments using time‐resolved femtosecond Vis/IR spectroscopy, time‐resolved X‐ray solution scattering, and time‐resolved serial crystallography at synchrotron and XFEL facilities. These detailed research protocols aim to ease collaboration across fields, acquaint early‐career researchers with essential concepts, and offer a comprehensive overview for established researchers.

**Abbreviations**
CC: correlation coefficient
DED: difference electron density
EMBO: European Molecular Biology Organization
FEBS: Federation of European Biochemical Societies
PCA: principal component analysis
TR‐XSS: time‐resolved X‐ray solution scattering

Biological activity is governed not by a single static structure but by dynamic population shifts across multiple timescales [1]. This behavior is critical for the function, regulation, and interactions of proteins. Light is able to initiate a cascade of structural transitions in a certain group of proteins that are inherently light sensitive, and techniques such as time‐resolved spectroscopy and time‐resolved X‐ray methods can uniquely capture these light‐triggered protein dynamics. Time‐resolved spectroscopy has revolutionized protein science by revealing excited states and the kinetic landscape of light‐triggered intermediates, while time‐resolved X‐ray methods have generated atomic resolution models of structural intermediates and revealed conformational dynamics by providing snapshots of the dynamic processes in light‐sensitive biomolecules [2, 3, 4, 5, 6, 7, 8, 9]. Despite their individual strengths, neither technique on its own can fully unravel the complexity of biomolecular dynamics.

This special issue arises from the EMBO and FEBS workshops ‘Time‐resolved spectroscopy meets time‐resolved crystallography: The Future of Dynamic Photobiology’, which provide a forum that bridges the two fields, facilitates the exchange of ideas, and identifies common challenges in experimental design and data interpretation [10, 11]. Both fields are experimentally complex and rapidly evolving, resulting in a high entry barrier for interdisciplinary scientists and early‐career researchers alike. The issue features five research protocols that serve as practical roadmaps for the design and data analysis of time‐resolved studies on dynamic photobiological systems.

In this issue, Zahn and Heyne describe a polarization‐resolved femtosecond Vis/IR spectroscopy setup optimized for detecting weak transient absorption signals from photoactive biological samples, along with an overview of data analysis and interpretation [12]. The following two research protocols provide a framework for light‐triggered time‐resolved X‐ray solution scattering (TR‐XSS) experiments. TR‐XSS, though not yet widely used, is a synchrotron‐based technique that enables real‐time structural characterization of proteins under near‐native conditions, offering access to dynamic and transient conformational changes in solution. Within this framework, complementary aspects of the experimental workflow are addressed by two protocols. Specifically, the research protocol by Matteo Levantino provides a step‐by‐step methodology to collect TR‐XSS data in well‐defined photoexcitation conditions that avoid nonlinear effects induced by an excessive laser peak power density [13]. In parallel, Magnus Andersson and coworkers present a practical pipeline for light‐triggered TR‐XSS experiments that spans data collection, data processing, kinetic analysis, and structural refinement, with accompanying Python scripts for implementation and analysis [14].

The issue then turns to time‐resolved serial crystallography, where accurate determination of light‐state occupancies and refinement of low‐occupancy intermediates remain major analytical challenges. Briony A. Yorke and coworkers present a protocol that exploits the inherent oversampling of serial crystallographic data through batch resampling and principal component analysis (PCA) [15]. This approach provides insight into the significance of small conformational changes in proteins along a reaction time course. When combined with extrapolation of structure factor amplitudes, the method helps further in the identification of low‐occupancy intermediates. A practical workflow for batch resampling, scaling, and clustering is provided together with guidelines for the effective use of open‐source software including RoPE and Xtrapol8. Complimentary to this, Manuel Maestre‐Reyna and coworkers present an efficient tool—the difference electron density correlation coefficient real space refinement algorithm, dFoCC—to refine local structural changes in a selected subset of chemical entities [16]. This approach uses the correlation coefficient (CC) between the observed and calculated difference electron density (DED) map using the light adapted versus dark coordinates as a quality metric. Illustrative examples are presented in all cases to showcase the broad applicability of the protocols to diverse protein systems triggered by light.

We welcome this ‘In the Limelight’ issue, which presents a collection of detailed and accessible workflows covering experimental design, data acquisition, and data analysis that address key experimental and analytical challenges in the field of Dynamic Photobiology. We hope that these research protocols will ease collaboration across fields, contribute to lowering the entry barrier for early‐career researchers, and serve as a practical resource for both early career and established researchers.

## Conflict of interest

The authors declare no conflict of interest.

## Author contributions

SMK, AR, and BAY wrote the editorial.
